# Supplementary material for: Built environment profiles for Latin American urban settings: The SALURBAL study
Source: PLoS One. 2021 Oct 26;16(10):e0257528. doi: 10.1371/journal.pone.0257528 (PMC8547632; doi:10.1371/journal.pone.0257528)
Supplement: S2 Table — (DOCX) [file pone.0257528.s002.docx]

| **S2 Table: City profiles and conditional probabilities** | | | | | |
| --- | --- | --- | --- | --- | --- |
|  | | **Urban Landscape** | | **Street Design** | |
| **Country** | **City name** | **Profile label** | **Conditional probability** | **Profile label** | **Conditional probability** |
| Argentina | Bahia Blanca | Proximate stones | 1,00 | Semi-hyperbolic grid | 0,98 |
| Argentina | Buenos Aires | Contiguous large inkblots | 1,00 | Spiderweb | 1,00 |
| Argentina | Catamarca | Proximate stones | 1,00 | Semi-hyperbolic grid | 0,99 |
| Argentina | Comodoro Rivadavia | Scattered pixels | 1,00 | Labyrinth | 0,98 |
| Argentina | Concordia | Scattered pixels | 1,00 | Labyrinth | 1,00 |
| Argentina | Cordoba | Contiguous large inkblots | 1,00 | Spiderweb | 0,99 |
| Argentina | Corrientes | Proximate inkblots | 1,00 | Spiderweb | 0,97 |
| Argentina | Formosa | Scattered pixels | 0,86 | Labyrinth | 1,00 |
| Argentina | Jujuy | Scattered pixels | 0,86 | Semi-hyperbolic grid | 0,87 |
| Argentina | La Rioja | Proximate stones | 0,75 | Labyrinth | 1,00 |
| Argentina | Mar del Plata | Proximate inkblots | 1,00 | Spiderweb | 0,97 |
| Argentina | Mendoza | Proximate inkblots | 1,00 | Labyrinth | 0,99 |
| Argentina | Neuquen-Plottier-Cipolletti | Proximate inkblots | 1,00 | Labyrinth | 1,00 |
| Argentina | Parana | Scattered pixels | 0,89 | Labyrinth | 0,99 |
| Argentina | Posadas | Proximate stones | 0,65 | Spiderweb | 0,56 |
| Argentina | Rawson-Trelew | Scattered pixels | 0,98 | Labyrinth | 1,00 |
| Argentina | Resistencia | Proximate stones | 0,93 | Labyrinth | 0,91 |
| Argentina | Rio Cuarto | Scattered pixels | 1,00 | Labyrinth | 1,00 |
| Argentina | Rio Gallegos | Scattered pixels | 1,00 | Labyrinth | 1,00 |
| Argentina | Rosario | Proximate inkblots | 1,00 | Semi-hyperbolic grid | 0,98 |
| Argentina | Salta | Proximate stones | 0,94 | Semi-hyperbolic grid | 0,99 |
| Argentina | San Carlos de Bariloche | Scattered pixels | 0,93 | Labyrinth | 1,00 |
| Argentina | San Juan | Proximate inkblots | 1,00 | Semi-hyperbolic grid | 0,95 |
| Argentina | San Luis | Scattered pixels | 1,00 | Labyrinth | 1,00 |
| Argentina | San Miguel de Tucuman-Tafi Viejo | Proximate inkblots | 1,00 | Semi-hyperbolic grid | 0,99 |
| Argentina | San Nicolas de los Arroyos | Proximate stones | 1,00 | Semi-hyperbolic grid | 0,99 |
| Argentina | San Rafael | Scattered pixels | 1,00 | Labyrinth | 1,00 |
| Argentina | Santa Fe | Proximate stones | 0,88 | Semi-hyperbolic grid | 0,98 |
| Argentina | Santa Rosa | Scattered pixels | 0,91 | Labyrinth | 1,00 |
| Argentina | Santiago del Estero | Proximate stones | 0,97 | Labyrinth | 1,00 |
| Argentina | Tandil | Scattered pixels | 1,00 | Labyrinth | 1,00 |
| Argentina | Villa Mercedes | Scattered pixels | 1,00 | Labyrinth | 1,00 |
| Brasil | Alagoinhas | Proximate stones | 1,00 | Semi-hyperbolic grid | 0,87 |
| Brasil | Anapolis | Proximate inkblots | 0,59 | Semi-hyperbolic grid | 0,99 |
| Brasil | Angra dos Reis | Proximate stones | 0,99 | Hyperbolic grid | 1,00 |
| Brasil | Apucarana | Proximate stones | 1,00 | Semi-hyperbolic grid | 0,82 |
| Brasil | Aracaju | Proximate inkblots | 0,97 | Spiderweb | 1,00 |
| Brasil | Aracatuba | Proximate stones | 1,00 | Semi-hyperbolic grid | 0,99 |
| Brasil | Araguaina | Proximate stones | 0,69 | Labyrinth | 1,00 |
| Brasil | Araguari | Scattered pixels | 0,95 | Labyrinth | 1,00 |
| Brasil | Arapiraca | Proximate stones | 1,00 | Spiderweb | 1,00 |
| Brasil | Arapongas | Proximate stones | 1,00 | Semi-hyperbolic grid | 0,99 |
| Brasil | Araraquara | Proximate stones | 1,00 | Semi-hyperbolic grid | 0,98 |
| Brasil | Araras | Proximate stones | 1,00 | Semi-hyperbolic grid | 0,99 |
| Brasil | Araruama | Proximate inkblots | 0,71 | Spiderweb | 0,91 |
| Brasil | Atibaia | Proximate stones | 0,96 | Hyperbolic grid | 0,86 |
| Brasil | Balneario Camboriu | Proximate stones | 0,99 | Spiderweb | 1,00 |
| Brasil | Barbacena | Proximate stones | 1,00 | Hyperbolic grid | 1,00 |
| Brasil | Barreiras | Scattered pixels | 1,00 | Labyrinth | 1,00 |
| Brasil | Barretos | Proximate stones | 0,97 | Hyperbolic grid | 0,77 |
| Brasil | Bauru | Proximate inkblots | 1,00 | Spiderweb | 1,00 |
| Brasil | Belem | Proximate inkblots | 0,54 | Semi-hyperbolic grid | 0,84 |
| Brasil | Belo Horizonte | Contiguous large inkblots | 1,00 | Spiderweb | 1,00 |
| Brasil | Bento Goncalves | Proximate stones | 1,00 | Hyperbolic grid | 1,00 |
| Brasil | Birigui | Proximate stones | 1,00 | Semi-hyperbolic grid | 0,98 |
| Brasil | Blumenau | Proximate inkblots | 1,00 | Hyperbolic grid | 1,00 |
| Brasil | Boa Vista | Proximate stones | 0,97 | Labyrinth | 1,00 |
| Brasil | Botucatu | Proximate stones | 0,81 | Semi-hyperbolic grid | 0,51 |
| Brasil | Braganca Paulista | Proximate stones | 1,00 | Semi-hyperbolic grid | 0,96 |
| Brasil | Brasilia | Contiguous large inkblots | 1,00 | Semi-hyperbolic grid | 0,98 |
| Brasil | Brusque | Proximate stones | 0,92 | Hyperbolic grid | 0,99 |
| Brasil | Cabo Frio | Proximate stones | 1,00 | Spiderweb | 0,97 |
| Brasil | Cachoeiro de Itapemirim | Proximate stones | 0,89 | Hyperbolic grid | 1,00 |
| Brasil | Campina Grande | Proximate stones | 1,00 | Semi-hyperbolic grid | 0,99 |
| Brasil | Campinas | Contiguous large inkblots | 1,00 | Spiderweb | 1,00 |
| Brasil | Campo Grande | Proximate inkblots | 1,00 | Semi-hyperbolic grid | 0,81 |
| Brasil | Campos dos Goytacazes | Proximate stones | 0,71 | Labyrinth | 0,93 |
| Brasil | Caraguatatuba | Proximate stones | 1,00 | Semi-hyperbolic grid | 0,95 |
| Brasil | Caruaru | Proximate stones | 1,00 | Semi-hyperbolic grid | 0,93 |
| Brasil | Cascavel | Proximate stones | 0,96 | Semi-hyperbolic grid | 0,96 |
| Brasil | Castanhal | Proximate stones | 1,00 | Semi-hyperbolic grid | 0,98 |
| Brasil | Catanduva | Proximate stones | 0,98 | Spiderweb | 1,00 |
| Brasil | Caxias | Scattered pixels | 0,95 | Labyrinth | 1,00 |
| Brasil | Caxias do Sul | Proximate stones | 0,97 | Hyperbolic grid | 0,91 |
| Brasil | Chapeco | Proximate stones | 1,00 | Spiderweb | 1,00 |
| Brasil | Conselheiro Lafaiete | Proximate stones | 1,00 | Semi-hyperbolic grid | 0,93 |
| Brasil | Criciuma | Proximate stones | 0,94 | Semi-hyperbolic grid | 0,71 |
| Brasil | Cuiaba | Proximate inkblots | 1,00 | Semi-hyperbolic grid | 0,99 |
| Brasil | Curitiba | Contiguous large inkblots | 1,00 | Spiderweb | 0,90 |
| Brasil | Divinopolis | Proximate stones | 0,99 | Spiderweb | 0,58 |
| Brasil | Dourados | Proximate stones | 1,00 | Labyrinth | 0,99 |
| Brasil | Feira de Santana | Proximate inkblots | 0,83 | Semi-hyperbolic grid | 0,98 |
| Brasil | Florianopolis | Proximate inkblots | 1,00 | Semi-hyperbolic grid | 0,81 |
| Brasil | Fortaleza | Contiguous large inkblots | 1,00 | Spiderweb | 1,00 |
| Brasil | Foz do Iguacu | Proximate inkblots | 1,00 | Spiderweb | 0,70 |
| Brasil | Franca | Proximate inkblots | 1,00 | Spiderweb | 1,00 |
| Brasil | Garanhuns | Proximate stones | 0,78 | Semi-hyperbolic grid | 0,98 |
| Brasil | Goiania | Contiguous large inkblots | 1,00 | Spiderweb | 1,00 |
| Brasil | Governador Valadares | Proximate stones | 0,74 | Labyrinth | 0,93 |
| Brasil | Guarapari | Proximate stones | 1,00 | Hyperbolic grid | 0,95 |
| Brasil | Guarapuava | Proximate stones | 0,99 | Labyrinth | 1,00 |
| Brasil | Guaratingueta | Proximate stones | 0,95 | Semi-hyperbolic grid | 0,93 |
| Brasil | Ilheus | Scattered pixels | 0,71 | Labyrinth | 0,95 |
| Brasil | Imperatriz | Scattered pixels | 0,96 | Labyrinth | 0,90 |
| Brasil | Ipatinga | Proximate stones | 1,00 | Hyperbolic grid | 0,97 |
| Brasil | Itabira | Scattered pixels | 0,89 | Hyperbolic grid | 0,97 |
| Brasil | Itabuna | Proximate stones | 1,00 | Semi-hyperbolic grid | 0,98 |
| Brasil | Itajai | Proximate stones | 0,72 | Spiderweb | 1,00 |
| Brasil | Itapetininga | Scattered pixels | 1,00 | Labyrinth | 0,97 |
| Brasil | Jaragua do Sul | Proximate stones | 0,74 | Hyperbolic grid | 1,00 |
| Brasil | Jau | Proximate stones | 1,00 | Semi-hyperbolic grid | 0,99 |
| Brasil | Jequie | Scattered pixels | 0,96 | Labyrinth | 0,99 |
| Brasil | Ji-Parana | Proximate stones | 0,90 | Labyrinth | 1,00 |
| Brasil | Joao Pessoa | Proximate inkblots | 1,00 | Spiderweb | 1,00 |
| Brasil | Joinville | Proximate inkblots | 1,00 | Semi-hyperbolic grid | 0,95 |
| Brasil | Juazeiro do Norte | Proximate stones | 1,00 | Semi-hyperbolic grid | 0,99 |
| Brasil | Juiz de Fora | Proximate stones | 1,00 | Hyperbolic grid | 1,00 |
| Brasil | Jundiai | Proximate inkblots | 1,00 | Spiderweb | 1,00 |
| Brasil | Lages | Proximate stones | 0,70 | Labyrinth | 0,99 |
| Brasil | Limeira | Proximate inkblots | 1,00 | Spiderweb | 0,88 |
| Brasil | Linhares | Scattered pixels | 1,00 | Labyrinth | 1,00 |
| Brasil | Londrina | Proximate stones | 0,55 | Semi-hyperbolic grid | 0,99 |
| Brasil | Macae | Proximate stones | 1,00 | Hyperbolic grid | 0,93 |
| Brasil | Macapa | Proximate stones | 0,99 | Labyrinth | 1,00 |
| Brasil | Maceio | Proximate inkblots | 1,00 | Spiderweb | 1,00 |
| Brasil | Manaus | Proximate inkblots | 0,86 | Labyrinth | 0,94 |
| Brasil | Maraba | Scattered pixels | 1,00 | Labyrinth | 1,00 |
| Brasil | Marilia | Proximate stones | 0,98 | Semi-hyperbolic grid | 0,99 |
| Brasil | Maringa | Proximate inkblots | 1,00 | Spiderweb | 0,99 |
| Brasil | Mogi Guacu | Proximate stones | 1,00 | Semi-hyperbolic grid | 0,99 |
| Brasil | Montes Claros | Proximate stones | 0,97 | Labyrinth | 0,81 |
| Brasil | Mossoro | Proximate stones | 1,00 | Semi-hyperbolic grid | 0,98 |
| Brasil | Natal | Proximate inkblots | 1,00 | Spiderweb | 1,00 |
| Brasil | Nova Friburgo | Proximate stones | 0,72 | Hyperbolic grid | 1,00 |
| Brasil | Ourinhos | Proximate stones | 1,00 | Spiderweb | 0,95 |
| Brasil | Palmas | Proximate stones | 0,81 | Semi-hyperbolic grid | 0,94 |
| Brasil | Paranagua | Proximate stones | 1,00 | Semi-hyperbolic grid | 0,88 |
| Brasil | Parauapebas | Scattered pixels | 1,00 | Labyrinth | 1,00 |
| Brasil | Parnaiba | Proximate stones | 1,00 | Semi-hyperbolic grid | 0,79 |
| Brasil | Parobe | Proximate stones | 1,00 | Hyperbolic grid | 1,00 |
| Brasil | Passo Fundo | Proximate stones | 1,00 | Semi-hyperbolic grid | 0,91 |
| Brasil | Passos | Scattered pixels | 0,73 | Labyrinth | 0,93 |
| Brasil | Patos de Minas | Scattered pixels | 1,00 | Labyrinth | 0,99 |
| Brasil | Pelotas | Proximate stones | 0,97 | Hyperbolic grid | 0,98 |
| Brasil | Petrolina | Scattered pixels | 1,00 | Labyrinth | 1,00 |
| Brasil | Petropolis | Proximate stones | 1,00 | Hyperbolic grid | 1,00 |
| Brasil | Piracicaba | Proximate inkblots | 0,96 | Semi-hyperbolic grid | 0,99 |
| Brasil | Pocos de Caldas | Proximate stones | 1,00 | Semi-hyperbolic grid | 0,95 |
| Brasil | Ponta Grossa | Proximate inkblots | 0,82 | Semi-hyperbolic grid | 0,95 |
| Brasil | Porto Alegre | Contiguous large inkblots | 1,00 | Spiderweb | 1,00 |
| Brasil | Porto Seguro | Scattered pixels | 0,93 | Labyrinth | 0,99 |
| Brasil | Porto Velho | Proximate inkblots | 0,98 | Labyrinth | 1,00 |
| Brasil | Pouso Alegre | Proximate stones | 1,00 | Semi-hyperbolic grid | 0,98 |
| Brasil | Presidente Prudente | Proximate stones | 1,00 | Spiderweb | 0,57 |
| Brasil | Recife | Contiguous large inkblots | 1,00 | Spiderweb | 1,00 |
| Brasil | Resende | Proximate stones | 0,76 | Hyperbolic grid | 1,00 |
| Brasil | Ribeirao Preto | Proximate inkblots | 1,00 | Spiderweb | 1,00 |
| Brasil | Rio Branco | Proximate stones | 0,98 | Labyrinth | 1,00 |
| Brasil | Rio Claro | Proximate stones | 0,99 | Semi-hyperbolic grid | 0,90 |
| Brasil | Rio das Ostras | Proximate stones | 1,00 | Semi-hyperbolic grid | 0,98 |
| Brasil | Rio de Janeiro | Contiguous large inkblots | 1,00 | Spiderweb | 1,00 |
| Brasil | Rio Grande | Scattered pixels | 0,94 | Labyrinth | 1,00 |
| Brasil | Rio Verde | Scattered pixels | 1,00 | Labyrinth | 1,00 |
| Brasil | Rondonopolis | Proximate stones | 1,00 | Labyrinth | 0,99 |
| Brasil | Salvador | Proximate inkblots | 0,94 | Spiderweb | 1,00 |
| Brasil | Santa Cruz do Sul | Proximate stones | 1,00 | Hyperbolic grid | 1,00 |
| Brasil | Santa Maria | Proximate stones | 1,00 | Hyperbolic grid | 0,59 |
| Brasil | Santarem | Scattered pixels | 1,00 | Labyrinth | 1,00 |
| Brasil | Santos | Proximate inkblots | 1,00 | Spiderweb | 1,00 |
| Brasil | Sao Carlos | Proximate stones | 1,00 | Semi-hyperbolic grid | 0,99 |
| Brasil | Sao Jose do Rio Preto | Proximate inkblots | 1,00 | Spiderweb | 1,00 |
| Brasil | Sao Jose dos Campos | Proximate inkblots | 0,93 | Semi-hyperbolic grid | 0,65 |
| Brasil | Sao Luis | Proximate inkblots | 0,94 | Spiderweb | 1,00 |
| Brasil | Sao Paulo | Contiguous large inkblots | 1,00 | Spiderweb | 1,00 |
| Brasil | Sertaozinho | Proximate stones | 1,00 | Semi-hyperbolic grid | 0,92 |
| Brasil | Sete Lagoas | Proximate inkblots | 1,00 | Spiderweb | 0,90 |
| Brasil | Sobral | Scattered pixels | 1,00 | Labyrinth | 0,97 |
| Brasil | Sorocaba | Proximate inkblots | 1,00 | Spiderweb | 1,00 |
| Brasil | Tatui | Proximate stones | 1,00 | Semi-hyperbolic grid | 0,97 |
| Brasil | Taubate | Proximate stones | 1,00 | Semi-hyperbolic grid | 0,99 |
| Brasil | Teixeira de Freitas | Proximate stones | 0,93 | Semi-hyperbolic grid | 0,82 |
| Brasil | Teofilo Otoni | Scattered pixels | 1,00 | Hyperbolic grid | 0,88 |
| Brasil | Teresina | Proximate inkblots | 1,00 | Semi-hyperbolic grid | 0,99 |
| Brasil | Teresopolis | Proximate stones | 1,00 | Hyperbolic grid | 1,00 |
| Brasil | Toledo | Proximate stones | 0,98 | Hyperbolic grid | 1,00 |
| Brasil | Tubarao | Proximate stones | 1,00 | Semi-hyperbolic grid | 0,90 |
| Brasil | Uberaba | Proximate stones | 1,00 | Labyrinth | 0,99 |
| Brasil | Uberlandia | Proximate inkblots | 1,00 | Semi-hyperbolic grid | 0,99 |
| Brasil | Uruguaiana | Scattered pixels | 1,00 | Labyrinth | 1,00 |
| Brasil | Varginha | Proximate stones | 1,00 | Semi-hyperbolic grid | 0,80 |
| Brasil | Vitoria | Proximate inkblots | 1,00 | Spiderweb | 1,00 |
| Brasil | Vitoria da Conquista | Proximate stones | 0,99 | Hyperbolic grid | 0,40 |
| Brasil | Vitoria de Santo Antao | Scattered pixels | 1,00 | Semi-hyperbolic grid | 0,96 |
| Brasil | Volta Redonda | Proximate stones | 0,99 | Hyperbolic grid | 1,00 |
| Chile | Antofagasta | Scattered pixels | 1,00 | Labyrinth | 1,00 |
| Chile | Arica | Scattered pixels | 0,88 | Labyrinth | 1,00 |
| Chile | Calama | Scattered pixels | 1,00 | Labyrinth | 1,00 |
| Chile | Chillan | Proximate stones | 1,00 | Semi-hyperbolic grid | 0,96 |
| Chile | Concepcion | Proximate stones | 0,94 | Spiderweb | 0,82 |
| Chile | Copiapo | Scattered pixels | 1,00 | Labyrinth | 1,00 |
| Chile | Curico | Proximate stones | 0,69 | Semi-hyperbolic grid | 0,49 |
| Chile | Iquique | Scattered pixels | 1,00 | Labyrinth | 0,99 |
| Chile | La Serena-Coquimbo | Scattered pixels | 0,87 | Semi-hyperbolic grid | 0,83 |
| Chile | Los Angeles | Proximate inkblots | 0,90 | Hyperbolic grid | 0,63 |
| Chile | Osorno | Proximate stones | 1,00 | Semi-hyperbolic grid | 0,95 |
| Chile | Puerto Montt | Proximate stones | 0,99 | Semi-hyperbolic grid | 0,77 |
| Chile | Punta Arenas | Scattered pixels | 0,98 | Labyrinth | 1,00 |
| Chile | Quillota | Proximate stones | 1,00 | Semi-hyperbolic grid | 0,93 |
| Chile | Rancagua | Proximate stones | 0,79 | Labyrinth | 0,77 |
| Chile | San Antonio | Proximate stones | 0,96 | Semi-hyperbolic grid | 0,95 |
| Chile | Santiago | Contiguous large inkblots | 1,00 | Spiderweb | 1,00 |
| Chile | Talca | Proximate inkblots | 1,00 | Spiderweb | 1,00 |
| Chile | Temuco | Proximate stones | 1,00 | Semi-hyperbolic grid | 0,75 |
| Chile | Valdivia | Proximate stones | 0,55 | Hyperbolic grid | 0,91 |
| Chile | Valparaiso-Vina del Mar | Proximate stones | 1,00 | Spiderweb | 1,00 |
| Colombia | Apartado | Scattered pixels | 1,00 | Labyrinth | 0,92 |
| Colombia | Armenia | Proximate stones | 1,00 | Spiderweb | 0,97 |
| Colombia | Barrancabermeja | Scattered pixels | 0,56 | Labyrinth | 0,98 |
| Colombia | Barranquilla | Proximate inkblots | 0,99 | Spiderweb | 1,00 |
| Colombia | Bogota | Contiguous large inkblots | 1,00 | Spiderweb | 1,00 |
| Colombia | Bucaramanga | Proximate stones | 1,00 | Hyperbolic grid | 1,00 |
| Colombia | Buenaventura | Scattered pixels | 1,00 | Hyperbolic grid | 0,58 |
| Colombia | Buga | Scattered pixels | 1,00 | Labyrinth | 1,00 |
| Colombia | Cali | Proximate inkblots | 1,00 | Spiderweb | 1,00 |
| Colombia | Cartagena | Proximate inkblots | 1,00 | Spiderweb | 1,00 |
| Colombia | Cartago | Scattered pixels | 0,59 | Semi-hyperbolic grid | 0,89 |
| Colombia | Cucuta | Proximate stones | 1,00 | Semi-hyperbolic grid | 0,98 |
| Colombia | Duitama | Scattered pixels | 0,91 | Hyperbolic grid | 1,00 |
| Colombia | Florencia | Scattered pixels | 1,00 | Labyrinth | 0,88 |
| Colombia | Fusagasuga | Proximate stones | 1,00 | Semi-hyperbolic grid | 0,98 |
| Colombia | Girardot | Proximate stones | 1,00 | Semi-hyperbolic grid | 0,95 |
| Colombia | Ibague | Proximate stones | 0,94 | Hyperbolic grid | 1,00 |
| Colombia | Manizales | Scattered pixels | 0,95 | Hyperbolic grid | 1,00 |
| Colombia | Medellin | Proximate inkblots | 0,96 | Spiderweb | 1,00 |
| Colombia | Monteria | Scattered pixels | 1,00 | Labyrinth | 0,99 |
| Colombia | Neiva | Proximate stones | 0,99 | Labyrinth | 0,95 |
| Colombia | Palmira | Proximate stones | 1,00 | Semi-hyperbolic grid | 0,89 |
| Colombia | Pasto | Scattered pixels | 0,88 | Hyperbolic grid | 0,83 |
| Colombia | Pereira | Proximate stones | 1,00 | Hyperbolic grid | 1,00 |
| Colombia | Popayan | Proximate stones | 1,00 | Semi-hyperbolic grid | 0,62 |
| Colombia | Quibdo | Scattered pixels | 1,00 | Labyrinth | 0,93 |
| Colombia | Riohacha | Scattered pixels | 1,00 | Labyrinth | 1,00 |
| Colombia | Santa Marta | Scattered pixels | 0,97 | Labyrinth | 1,00 |
| Colombia | Sincelejo | Proximate stones | 1,00 | Spiderweb | 1,00 |
| Colombia | Sogamoso | Proximate stones | 1,00 | Hyperbolic grid | 0,99 |
| Colombia | Tulua | Scattered pixels | 0,99 | Hyperbolic grid | 1,00 |
| Colombia | Tunja | Scattered pixels | 1,00 | Semi-hyperbolic grid | 0,97 |
| Colombia | Valledupar | Scattered pixels | 0,95 | Labyrinth | 1,00 |
| Colombia | Villavicencio | Proximate stones | 1,00 | Semi-hyperbolic grid | 0,93 |
| Colombia | Yopal | Scattered pixels | 1,00 | Labyrinth | 0,99 |
| Costa Rica | San Jose | Contiguous large inkblots | 1,00 | Spiderweb | 0,98 |
| El Salvador | San Miguel | Proximate stones | 1,00 | Semi-hyperbolic grid | 0,99 |
| El Salvador | San Salvador | Proximate inkblots | 1,00 | Spiderweb | 1,00 |
| El Salvador | Santa Ana | Proximate stones | 1,00 | Spiderweb | 0,95 |
| Guatemala | Escuintla | Scattered pixels | 0,57 | Hyperbolic grid | 1,00 |
| Guatemala | Guatemala City | Proximate inkblots | 0,94 | Spiderweb | 1,00 |
| Guatemala | Quetzaltenango | Proximate inkblots | 1,00 | Spiderweb | 1,00 |
| Mexico | Acapulco de Juarez | Proximate inkblots | 1,00 | Hyperbolic grid | 0,97 |
| Mexico | Acayucan | Scattered pixels | 1,00 | Labyrinth | 0,99 |
| Mexico | Acuna | Scattered pixels | 1,00 | Labyrinth | 1,00 |
| Mexico | Aguascalientes | Proximate inkblots | 1,00 | Spiderweb | 0,99 |
| Mexico | Campeche | Scattered pixels | 0,98 | Labyrinth | 1,00 |
| Mexico | Cancun | Proximate inkblots | 0,97 | Semi-hyperbolic grid | 0,98 |
| Mexico | Celaya | Proximate inkblots | 1,00 | Semi-hyperbolic grid | 0,99 |
| Mexico | Chetumal | Scattered pixels | 1,00 | Labyrinth | 1,00 |
| Mexico | Chihuahua | Proximate inkblots | 1,00 | Labyrinth | 0,99 |
| Mexico | Chilpancingo | Scattered pixels | 1,00 | Labyrinth | 0,87 |
| Mexico | Ciudad del Carmen | Scattered pixels | 0,96 | Labyrinth | 1,00 |
| Mexico | Ciudad Valles | Scattered pixels | 0,86 | Labyrinth | 0,98 |
| Mexico | Coatzacoalcos | Proximate stones | 0,96 | Spiderweb | 1,00 |
| Mexico | Colima | Proximate stones | 1,00 | Semi-hyperbolic grid | 0,99 |
| Mexico | Cordoba | Proximate stones | 1,00 | Spiderweb | 0,98 |
| Mexico | Cuauhtemoc | Proximate inkblots | 0,97 | Labyrinth | 1,00 |
| Mexico | Cuautla | Proximate stones | 0,98 | Semi-hyperbolic grid | 0,78 |
| Mexico | Cuernavaca | Proximate inkblots | 0,99 | Spiderweb | 1,00 |
| Mexico | Culiacan | Proximate inkblots | 1,00 | Semi-hyperbolic grid | 0,95 |
| Mexico | Delicias | Proximate stones | 1,00 | Semi-hyperbolic grid | 0,89 |
| Mexico | Durango | Proximate inkblots | 0,99 | Labyrinth | 0,98 |
| Mexico | Ensenada | Scattered pixels | 0,90 | Labyrinth | 0,99 |
| Mexico | Fresnillo | Scattered pixels | 1,00 | Labyrinth | 1,00 |
| Mexico | Guadalajara | Contiguous large inkblots | 1,00 | Spiderweb | 1,00 |
| Mexico | Guanajuato | Scattered pixels | 1,00 | Hyperbolic grid | 1,00 |
| Mexico | Guaymas | Scattered pixels | 1,00 | Labyrinth | 1,00 |
| Mexico | Hermosillo | Proximate inkblots | 1,00 | Labyrinth | 0,99 |
| Mexico | Hidalgo del Parral | Scattered pixels | 1,00 | Labyrinth | 1,00 |
| Mexico | Iguala | Proximate stones | 1,00 | Labyrinth | 0,58 |
| Mexico | Irapuato | Proximate inkblots | 0,85 | Semi-hyperbolic grid | 0,99 |
| Mexico | Juarez | Proximate inkblots | 1,00 | Semi-hyperbolic grid | 0,80 |
| Mexico | La Paz | Scattered pixels | 1,00 | Labyrinth | 1,00 |
| Mexico | La Piedad | Scattered pixels | 1,00 | Labyrinth | 0,92 |
| Mexico | Leon | Proximate inkblots | 1,00 | Spiderweb | 1,00 |
| Mexico | Los Mochis | Proximate stones | 0,92 | Semi-hyperbolic grid | 0,86 |
| Mexico | Manzanillo | Proximate stones | 1,00 | Hyperbolic grid | 0,96 |
| Mexico | Matamoros | Proximate inkblots | 0,90 | Semi-hyperbolic grid | 0,71 |
| Mexico | Mazatlan | Proximate stones | 0,90 | Semi-hyperbolic grid | 0,97 |
| Mexico | Merida | Proximate inkblots | 0,96 | Spiderweb | 1,00 |
| Mexico | Mexicali | Proximate inkblots | 1,00 | Labyrinth | 0,99 |
| Mexico | Mexico City | Contiguous large inkblots | 1,00 | Spiderweb | 1,00 |
| Mexico | Minatitlan | Proximate stones | 0,96 | Labyrinth | 0,98 |
| Mexico | Monclova | Proximate stones | 0,97 | Labyrinth | 0,99 |
| Mexico | Monterrey | Contiguous large inkblots | 1,00 | Spiderweb | 1,00 |
| Mexico | Morelia | Proximate inkblots | 1,00 | Spiderweb | 0,99 |
| Mexico | Navojoa | Scattered pixels | 1,00 | Labyrinth | 0,97 |
| Mexico | Nogales | Proximate stones | 0,99 | Labyrinth | 0,90 |
| Mexico | Nuevo Laredo | Proximate stones | 0,97 | Semi-hyperbolic grid | 0,96 |
| Mexico | Oaxaca de Juarez | Proximate inkblots | 1,00 | Spiderweb | 1,00 |
| Mexico | Obregon | Proximate stones | 0,94 | Labyrinth | 0,93 |
| Mexico | Ocotlan | Scattered pixels | 1,00 | Semi-hyperbolic grid | 0,91 |
| Mexico | Orizaba | Proximate stones | 0,62 | Semi-hyperbolic grid | 0,97 |
| Mexico | Pachuca de Soto | Proximate inkblots | 1,00 | Semi-hyperbolic grid | 0,93 |
| Mexico | Piedras Negras | Proximate stones | 1,00 | Semi-hyperbolic grid | 0,98 |
| Mexico | Playa del Carmen | Scattered pixels | 0,92 | Labyrinth | 0,82 |
| Mexico | Poza Rica de Hidalgo | Proximate inkblots | 0,98 | Semi-hyperbolic grid | 0,94 |
| Mexico | Puebla de Zaragoza | Contiguous large inkblots | 1,00 | Spiderweb | 1,00 |
| Mexico | Puerto Vallarta | Proximate stones | 1,00 | Semi-hyperbolic grid | 0,94 |
| Mexico | Queretaro | Proximate inkblots | 1,00 | Spiderweb | 1,00 |
| Mexico | Reynosa | Proximate inkblots | 1,00 | Semi-hyperbolic grid | 0,93 |
| Mexico | Rio Verde | Scattered pixels | 1,00 | Labyrinth | 1,00 |
| Mexico | Salamanca | Proximate inkblots | 0,97 | Semi-hyperbolic grid | 0,96 |
| Mexico | Saltillo | Proximate inkblots | 1,00 | Labyrinth | 0,99 |
| Mexico | San Cristobal de las Casas | Proximate stones | 1,00 | Semi-hyperbolic grid | 0,81 |
| Mexico | San Francisco del Rincon | Proximate stones | 1,00 | Labyrinth | 0,78 |
| Mexico | San Juan Bautista Tuxtepec | Proximate stones | 0,91 | Labyrinth | 0,93 |
| Mexico | San Juan del Rio | Proximate stones | 1,00 | Semi-hyperbolic grid | 0,55 |
| Mexico | San Luis Potosi | Proximate inkblots | 1,00 | Spiderweb | 0,80 |
| Mexico | San Luis Rio Colorado | Proximate stones | 0,99 | Labyrinth | 1,00 |
| Mexico | Santo Domingo Tehuantepec | Scattered pixels | 1,00 | Semi-hyperbolic grid | 0,90 |
| Mexico | Tampico | Proximate inkblots | 0,99 | Semi-hyperbolic grid | 0,57 |
| Mexico | Tapachula | Proximate stones | 1,00 | Semi-hyperbolic grid | 0,95 |
| Mexico | Tecoman | Scattered pixels | 1,00 | Labyrinth | 0,96 |
| Mexico | Tehuacan | Proximate stones | 1,00 | Semi-hyperbolic grid | 0,98 |
| Mexico | Tepic | Proximate stones | 0,97 | Semi-hyperbolic grid | 0,96 |
| Mexico | Teziutlan | Proximate stones | 0,99 | Semi-hyperbolic grid | 0,58 |
| Mexico | Tianguistenco | Proximate stones | 1,00 | Hyperbolic grid | 0,94 |
| Mexico | Tijuana | Proximate inkblots | 1,00 | Semi-hyperbolic grid | 0,96 |
| Mexico | Tlaxcala | Proximate inkblots | 1,00 | Spiderweb | 1,00 |
| Mexico | Toluca | Contiguous large inkblots | 1,00 | Spiderweb | 0,96 |
| Mexico | Torreon | Proximate inkblots | 1,00 | Semi-hyperbolic grid | 0,99 |
| Mexico | Tula de Allende | Proximate stones | 0,97 | Hyperbolic grid | 0,97 |
| Mexico | Tulancingo de Bravo | Proximate stones | 1,00 | Semi-hyperbolic grid | 0,88 |
| Mexico | Tuxtla Gutierrez | Proximate inkblots | 1,00 | Semi-hyperbolic grid | 0,85 |
| Mexico | Uriangato | Proximate stones | 1,00 | Semi-hyperbolic grid | 0,99 |
| Mexico | Uruapan | Proximate stones | 1,00 | Semi-hyperbolic grid | 0,98 |
| Mexico | Veracruz | Proximate inkblots | 1,00 | Semi-hyperbolic grid | 0,80 |
| Mexico | Victoria | Proximate stones | 0,92 | Semi-hyperbolic grid | 0,98 |
| Mexico | Villahermosa | Proximate inkblots | 1,00 | Hyperbolic grid | 1,00 |
| Mexico | Xalapa | Proximate inkblots | 1,00 | Spiderweb | 1,00 |
| Mexico | Zacatecas | Proximate stones | 1,00 | Semi-hyperbolic grid | 0,97 |
| Mexico | Zamora | Proximate stones | 1,00 | Semi-hyperbolic grid | 0,88 |
| Nicaragua | Chinandega | Scattered pixels | 0,97 | Labyrinth | 1,00 |
| Nicaragua | Esteli | Scattered pixels | 0,94 | Labyrinth | 0,96 |
| Nicaragua | Leon | Proximate stones | 0,74 | Semi-hyperbolic grid | 0,88 |
| Nicaragua | Managua | Proximate inkblots | 1,00 | Spiderweb | 1,00 |
| Nicaragua | Masaya | Proximate inkblots | 0,99 | Semi-hyperbolic grid | 0,95 |
| Panama | Colon | Proximate stones | 1,00 | Hyperbolic grid | 0,99 |
| Panama | David | Proximate stones | 1,00 | Semi-hyperbolic grid | 0,85 |
| Panama | Panama City | Proximate inkblots | 1,00 | Semi-hyperbolic grid | 0,64 |
| Peru | Arequipa | Proximate inkblots | 1,00 | Semi-hyperbolic grid | 0,99 |
| Peru | Ayacucho | Proximate stones | 1,00 | Spiderweb | 1,00 |
| Peru | Cajamarca | Proximate stones | 1,00 | Hyperbolic grid | 1,00 |
| Peru | Chiclayo | Proximate stones | 1,00 | Spiderweb | 0,99 |
| Peru | Chimbote | Scattered pixels | 0,93 | Semi-hyperbolic grid | 0,86 |
| Peru | Chincha Alta | Scattered pixels | 0,92 | Labyrinth | 0,95 |
| Peru | Cusco | Proximate inkblots | 0,99 | Spiderweb | 1,00 |
| Peru | Huancayo | Proximate inkblots | 0,93 | Semi-hyperbolic grid | 0,89 |
| Peru | Huanuco | Scattered pixels | 0,95 | Hyperbolic grid | 1,00 |
| Peru | Huaraz | Scattered pixels | 0,96 | Hyperbolic grid | 1,00 |
| Peru | Ica | Scattered pixels | 0,97 | Labyrinth | 1,00 |
| Peru | Iquitos | Proximate stones | 0,71 | Labyrinth | 0,99 |
| Peru | Juliaca | Proximate stones | 1,00 | Semi-hyperbolic grid | 0,99 |
| Peru | Lima | Contiguous large inkblots | 1,00 | Spiderweb | 1,00 |
| Peru | Pisco | Scattered pixels | 0,98 | Semi-hyperbolic grid | 0,98 |
| Peru | Piura | Proximate stones | 1,00 | Labyrinth | 0,97 |
| Peru | Pucallpa | Proximate stones | 0,99 | Labyrinth | 1,00 |
| Peru | Puno | Scattered pixels | 0,97 | Semi-hyperbolic grid | 0,90 |
| Peru | Sullana | Scattered pixels | 1,00 | Labyrinth | 1,00 |
| Peru | Tacna | Proximate stones | 1,00 | Labyrinth | 0,97 |
| Peru | Tarapoto | Proximate stones | 1,00 | Hyperbolic grid | 1,00 |
| Peru | Trujillo | Proximate inkblots | 1,00 | Semi-hyperbolic grid | 0,79 |
| Peru | Tumbes | Proximate stones | 0,59 | Semi-hyperbolic grid | 0,99 |
