# Supplementary material for: Built environment profiles for Latin American urban settings: The SALURBAL study
Source: PLoS One. 2021 Oct 26;16(10):e0257528. doi: 10.1371/journal.pone.0257528 (PMC8547632; doi:10.1371/journal.pone.0257528)
Supplement: S1 Fig — A 1.5% of decrease in the Bayesian Information Criteria (BIC) function was used as stop criteria to determine the number of latent classes in each dimension. RE corresponds to relative entropy and PDBIC corresponds to the percentage of decreasing in BIC. (DOCX) [file pone.0257528.s004.docx]

**S3 Figure: Elbow method selection for determining the number of latent classes**


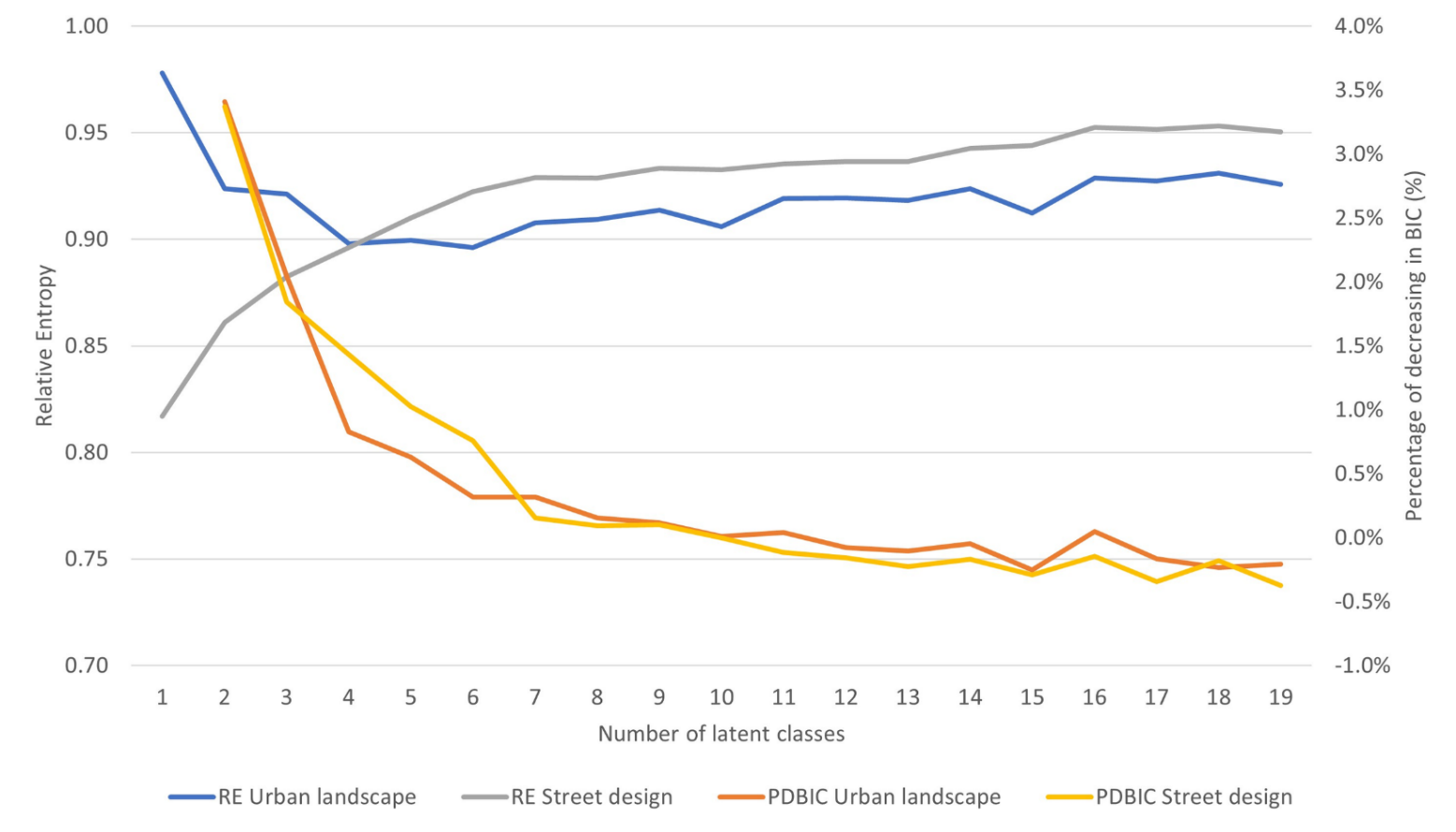


A 1.5% of decrease in the Bayesian Information Criteria (BIC) function was used as stop criteria to determine the number of latent classes in each dimension. RE corresponds to relative entropy and PDBIC corresponds to the percentage of decreasing in BIC.
