# Supplementary material for: Built environment profiles for Latin American urban settings: The SALURBAL study
Source: PLoS One. 2021 Oct 26;16(10):e0257528. doi: 10.1371/journal.pone.0257528 (PMC8547632; doi:10.1371/journal.pone.0257528)
Supplement: S1 Appendix — (DOCX) [file pone.0257528.s006.docx]

| **Appendix 1: Urban landscape and street design descriptives for Latin American countries** | | | | | | | | | | | | | | | | | | | | | | | | | | | | |
| --- | --- | --- | --- | --- | --- | --- | --- | --- | --- | --- | --- | --- | --- | --- | --- | --- | --- | --- | --- | --- | --- | --- | --- | --- | --- | --- | --- | --- |
| **Dimensions, subdimensions and metrics** | | | **Overall (n = 370)** | | **Argentina (n = 33)** | | **Brazil (n = 152)** | | **Chile (n = 21)** | | **Colombia (n = 35)** | | **Costa Rica (n = 1)** | | **El Salvador (n = 3)** | | **Guatemala (n = 3)** | | **Mexico (n = 92)** | | **Nicaragua (n = 5)** | | **Panama (n = 3)** | | **Peru (n = 23)** | | |  |
|  |  |  | P50 | (IQR) | P50 | (IQR) | P50 | (IQR) | P50 | (IQR) | P50 | (IQR) | P50 | (IQR) | P50 | (IQR) | P50 | (IQR) | P50 | (IQR) | P50 | (IQR) | P50 | (IQR) | P50 | (IQR) | |  |
| **Urban Landscape** | | | | | | | | | | | | | | | | | | | | | | | | | | |  | |
|  | **Fragmentation** | |  |  |  |  |  |  |  |  |  |  |  |  |  |  |  |  |  |  |  |  |  |  |  |  | |  |
|  |  | Number of Urban Patches (N) | 660,3 | (251.0;744.5) | 492,0 | (338.0;1070.0) | 400,0 | (259.5;646.3) | 420,0 | (262.0;576.0) | 212,0 | (129.5;325.0) | 2888,0 | NA | 419,0 | (358.0;756.5) | 512,0 | (364.5;1014.0) | 566,0 | (363.5;952.5) | 253,0 | (249.0;257.0) | 592,0 | (528.5;1192.0) | 173,0 | (96.5;302.5) | |  |
|  |  | Patch Density (N/km2) | 0,37 | (0.12;0.56) | 0,17 | (0.1;0.3) | 0,35 | (0.1;0.6) | 0,30 | (0.1;0.5) | 0,23 | (0.1;0.5) | 0,93 | NA | 0,73 | (0.7;1.0) | 0,85 | (0.6;1.7) | 0,31 | (0.2;0.6) | 0,31 | (0.2;1.2) | 0,53 | (0.5;0.6) | 0,14 | (0.1;0.3) | |  |
|  |  | Area-weighted Mean Patch Size | 4751,7 | (1076.4;3925.2) | 2196,9 | (1268.2;4446.8) | 1947,9 | (1251.9;3720.9) | 1569,8 | (1083.1;1994.7) | 1060,4 | (704.6;1874.7) | 17494,5 | NA | 1439,5 | (1089.3;4298.8) | 1674,8 | (1084.2;5345.7) | 2320,0 | (1278.6;5173.5) | 734,7 | (708.8;893.8) | 996,5 | (989.6;3518.2) | 1381,5 | (890.5;2881.9) | |  |
|  |  | Effective Mesh Size | 603,6 | (18.1;183.3) | 31,8 | (8.2;181.7) | 66,4 | (28.5;195.2) | 21,9 | (9.2;91.5) | 22,7 | (10.3;59.5) | 1941,2 | NA | 54,4 | (44.3;599.7) | 321,5 | (167.5;768.4) | 94,2 | (26.4;206.5) | 17,5 | (9.7;49.0) | 47,5 | (37.8;235.1) | 29,1 | (12.8;123.0) | |  |
|  | **Shape** | |  |  |  |  |  |  |  |  |  |  |  |  |  |  |  |  |  |  |  |  |  |  |  |  | |  |
|  |  | Area-weighted Mean Shape Index | 5,45 | (4.33;6.26) | 4,48 | (4.0;5.5) | 5,31 | (4.6;6.6) | 4,34 | (3.7;5.1) | 4,25 | (3.7;5.4) | 12,01 | NA | 4,47 | (4.2;6.1) | 6,40 | (5.3;7.6) | 5,32 | (4.5;6.6) | 3,99 | (3.9;4.4) | 6,84 | (5.8;6.9) | 4,75 | (4.3;5.8) | |  |
|  | **Isolation** | |  |  |  |  |  |  |  |  |  |  |  |  |  |  |  |  |  |  |  |  |  |  |  |  | |  |
|  |  | Area-weighted Mean Nearest Neighbor Distance (meters) | 95,9 | (72.3;102.8) | 83,0 | (74.4;120.7) | 80,3 | (71.5;94.5) | 81,9 | (73.0;101.1) | 88,6 | (75.5;116.9) | 65,4 | NA | 71,3 | (67.8;71.7) | 67,5 | (66.2;95.2) | 91,7 | (76.0;117.8) | 90,3 | (80.5;90.4) | 71,8 | (69.8;75.7) | 81,0 | (70.7;92.7) | |  |
| **Street design** | | | | | | | | | | | | | | | | | | | | | | | | | | |  | |
|  | **Street connectivity** | |  |  |  |  |  |  |  |  |  |  |  |  |  |  |  |  |  |  |  |  |  |  |  |  | |  |
|  |  | Street density | 1440,5 | (529.3;1956.9) | 591,4 | (296.3;1182.7) | 1556,2 | (787.9;2210.6) | 941,8 | (378.3;1671.4) | 1043,5 | (414.6;1730.9) | 2442,5 | NA | 2388,4 | (2018.0;3025.4) | 4075,9 | (2606.3;4337.9) | 964,3 | (559.0;1768.4) | 884,0 | (479.9;1903.1) | 1440,4 | (1087.6;1453.0) | 942,2 | (503.1;1537.9) | |  |
|  |  | Intersection density | 6,65 | (2.01;9.07) | 1,73 | (0.7;5.2) | 5,94 | (2.9;9.5) | 4,13 | (1.6;8.5) | 4,03 | (1.9;7.4) | 10,27 | NA | 10,70 | (8.8;14.6) | 20,42 | (11.9;22.2) | 4,05 | (1.9;8.2) | 3,20 | (2.1;6.5) | 4,95 | (3.4;5.3) | 3,52 | (2.4;8.8) | |  |
|  |  | Streets per node average | 2,98 | (2.85;3.13) | 3,21 | (3.2;3.3) | 3,01 | (2.8;3.1) | 2,86 | (2.8;2.9) | 2,93 | (2.9;3.1) | 2,56 | NA | 2,66 | (2.6;2.7) | 2,66 | (2.7;2.7) | 2,99 | (2.9;3.1) | 2,95 | (2.9;3.0) | 2,61 | (2.6;2.7) | 3,06 | (3.0;3.2) | |  |
|  | **Street Length** | |  |  |  |  |  |  |  |  |  |  |  |  |  |  |  |  |  |  |  |  |  |  |  |  | |  |
|  |  | Street length average | 149,5 | (118.6;163.8) | 164,9 | (138.8;216.2) | 138,1 | (122.1;169.1) | 123,8 | (115.8;150.3) | 121,7 | (100.4;135.4) | 133,1 | NA | 122,4 | (117.2;127.9) | 113,1 | (111.0;150.5) | 133,2 | (114.7;154.2) | 156,4 | (128.9;174.0) | 163,8 | (144.3;232.6) | 133,1 | (119.4;161.3) | |  |
|  | **Directness** | |  |  |  |  |  |  |  |  |  |  |  |  |  |  |  |  |  |  |  |  |  |  |  |  | |  |
|  |  | Circuity average | 1,07 | (1.04;1.09) | 1,05 | (1.0;1.1) | 1,07 | (1.0;1.1) | 1,09 | (1.1;1.1) | 1,09 | (1.1;1.1) | 1,12 | NA | 1,08 | (1.1;1.1) | 1,11 | (1.1;1.1) | 1,06 | (1.0;1.1) | 1,06 | (1.1;1.1) | 1,09 | (1.1;1.1) | 1,05 | (1.0;1.1) | |  |
| **Socio-economic and transport metrics** | | |  |  |  |  |  |  |  |  |  |  |  |  |  |  |  |  |  |  |  |  |  |  |  |  | |  |
|  |  | Proportion of households with piped water access | 94,60 | (93.6;99.3) | 92,6 | (89.5;95.0) | 97,6 | (92.8;98.5) | 95,7 | (93.7;97.7) | 87,4 | (73.0;90.0) | 99,3 | NA | 72,2 | (69.2;78.1) | 74,0 | (70.2;74.7) | 80,9 | (69.2;89.9) | 60,2 | (51.0;68.7) | 82,6 | (80.1;84.0) | 70,8 | (65.6;76.0) | |  |
|  |  | Proportion of households with more than 3 people per bedroom | 5,91 | (2.46;8.27) | 3,55 | (2.98;4.87) | 2,35 | (1.50;3.20) | 3,53 | (3.12;4.50) | 4,74 | (3.25;8.00) | 0,55 | NA | 13,61 | (12.99;14.11) | 19,06 | (18.11;22.65) | 8,45 | (6.63;11.64) | - | - | 4,10 | (3.63;4.57) | 13,14 | (11.99;16.80) | |  |
|  |  | Proportion of the population aged 25 or older who completed primary of above | 72,55 | (66.01;80.02) | 79,60 | (66.0;80.0) | 65,70 | (61.5;69.6) | 82,10 | (80.4;86.1) | 74,30 | (70.8;77.8) | 86,10 | NA | 57,30 | (54.2;63.6) | 54,70 | (52.1;59.8) | 79,50 | (74.4;83.0) | 81,70 | (80.3;83.4) | 91,00 | (89.1;91.4) | 75,70 | (72.2;77.8) | |  |
|  |  | Urban Travel Delay Index (SEI) | 0,2 | (0.08;0.20) | 0,17 | (0.11;0.27) | 0,11 | (0.08;0.19) | 0,08 | (0.04;0.13) | 0,15 | (0.08;0.2) | 0,04 | NA | 0,27 | (0.09;0.33) | 0,23 | (0.01;0.34) | 0,13 | (0.09;0.23) | 0,19 | (0.1;0.31) | 0,12 | (0.1;0.16) | 0,09 | (0.07;0.16) | |  |
| IQR: Interquartile range. NA: For Costa Rica we have only one city so the IQR is non applicable | | | | | | | | | | | | | | | | | | | | | | | | | | | | |
