# Supplementary material for: Built environment profiles for Latin American urban settings: The SALURBAL study
Source: PLoS One. 2021 Oct 26;16(10):e0257528. doi: 10.1371/journal.pone.0257528 (PMC8547632; doi:10.1371/journal.pone.0257528)
Supplement: S2 Appendix — (DOCX) [file pone.0257528.s007.docx]

**Appendix 3: Elbow method selection for determining the number of latent classes**

| a)  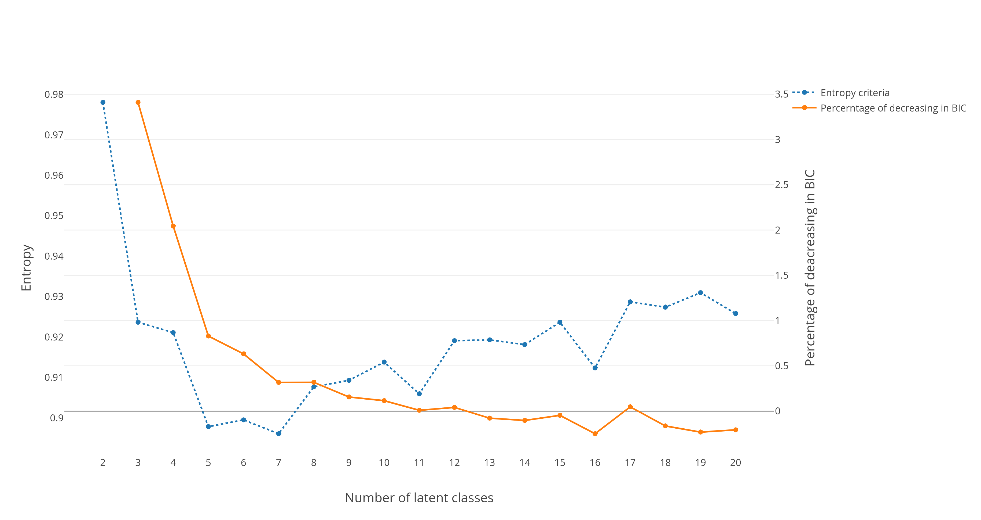 |
| --- |
| b)  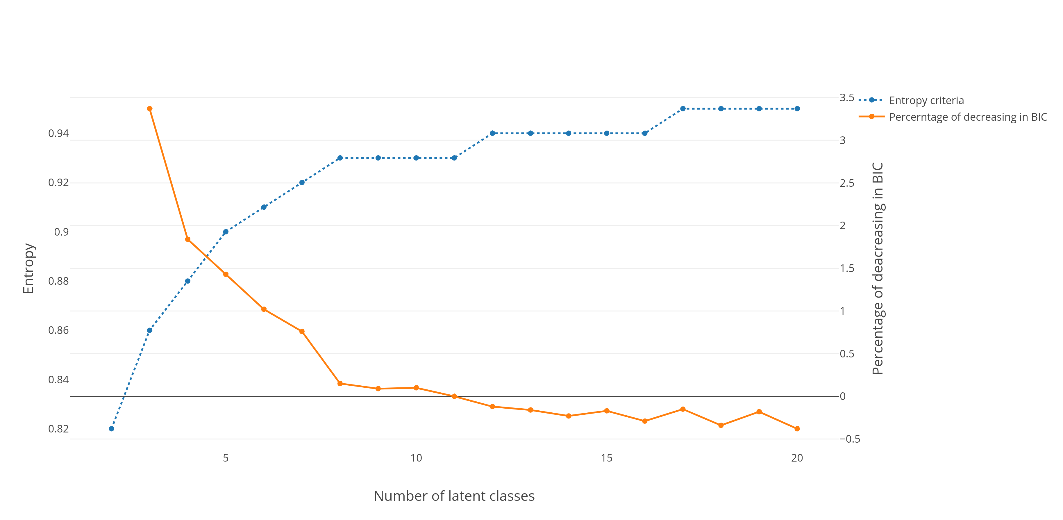 |

A 1.5% of decrease in BIC function was used as stop criteria to determine the number of latent classes. a) urban landscape classes. b) street design classes.
