# Supplementary material for: Built environment profiles for Latin American urban settings: The SALURBAL study
Source: PLoS One. 2021 Oct 26;16(10):e0257528. doi: 10.1371/journal.pone.0257528 (PMC8547632; doi:10.1371/journal.pone.0257528)
Supplement: S3 Appendix — (DOCX) [file pone.0257528.s008.docx]

**Appendix 3: Distribution of conditional probabilities of the cities to belong to each profile**

| a)  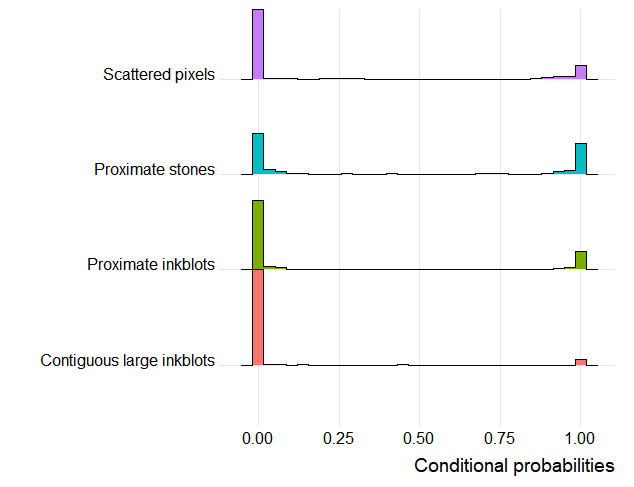 | b)  **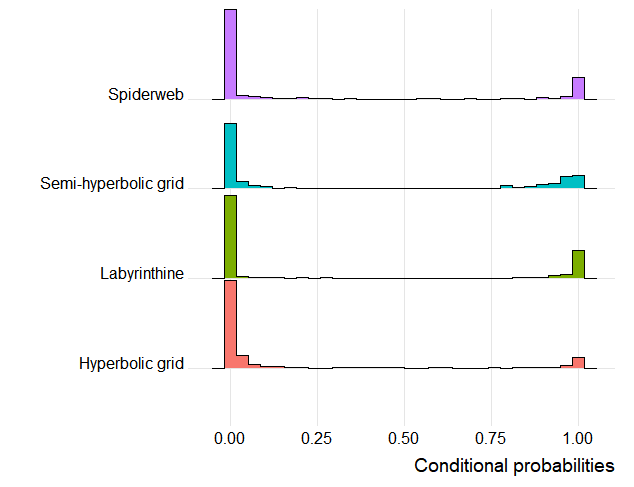** |
| --- | --- |

Distribution of the conditional probabilities of 370 cities is plotted in bars, where bigger bars reflect a higher proportion of cities. a) urban landscape profiles and b) street design profiles.
