# Supplementary material for: Built environment profiles for Latin American urban settings: The SALURBAL study
Source: PLoS One. 2021 Oct 26;16(10):e0257528. doi: 10.1371/journal.pone.0257528 (PMC8547632; doi:10.1371/journal.pone.0257528)
Supplement: S5 Appendix — (DOCX) [file pone.0257528.s010.docx]

| **Appendix 5: Type of colonies and foundation year for 70 cities with highest conditional probabilities in profiles** | | | | | |  |
| --- | --- | --- | --- | --- | --- | --- |
| **City name** | **Country** | **Total Area** | **Population density 2017** | **Type of colony** | **City year foundation** |  |
| Mexico City | Mexico | 7907,29 | 11537,13 | Prehispanic | 1325 |  |
| Quetzaltenango | Guatemala | 205,86 |  | Iberian | 1524 |  |
| Tecoman | Mexico | 1354,92 | 6825,98 | Iberian | 1527 |  |
| Puebla de Zaragoza | Mexico | 2699,87 | 6558,76 | Iberian | 1531 |  |
| Cartagena | Colombia | 840,59 | 15520,09 | Iberian | 1533 |  |
| Porto Seguro | Brasil | 2421,88 | 8936,71 | Iberian | 1534 |  |
| Lima | Peru | 3010,62 | 17654,11 | Iberian | 1535 |  |
| Buenos Aires | Argentina | 10004,46 | 7848,01 | Iberian | 1536 |  |
| Recife | Brasil | 1896,41 | 10675,42 | Iberian | 1537 |  |
| Bogota | Colombia | 3070,50 | 21441,27 | Iberian | 1538 |  |
| Morelia | Mexico | 1780,39 | 7750,61 | Iberian | 1541 |  |
| Riohacha | Colombia | 3097,58 | 11420,70 | Iberian | 1545 |  |
| Salvador | Brasil | 1747,38 | 15203,05 | Iberian | 1549 |  |
| Santiago del Estero | Argentina | 5868,63 | 6237,66 | Iberian | 1553 |  |
| Sao Paulo | Brasil | 5666,10 | 11427,63 | Iberian | 1554 |  |
| San Juan | Argentina | 1276,02 | 5073,54 | Iberian | 1562 |  |
| San Miguel de Tucuman-Tafi Viejo | Argentina | 3130,80 | 7014,70 | Local | 1565 |  |
| Santa Fe | Argentina | 2983,85 | 5472,22 | Iberian | 1573 |  |
| Leon | Mexico | 1768,89 | 9467,59 | Iberian | 1576 |  |
| Salta | Argentina | 3367,16 | 7470,89 | Iberian | 1582 |  |
| La Rioja | Argentina | 13152,44 | 5011,59 | Iberian | 1591 |  |
| San Luis | Argentina | 13086,74 | 5465,93 | Iberian | 1592 |  |
| Jujuy | Argentina | 2536,76 | 8484,95 | Iberian | 1593 |  |
| Monterrey | Mexico | 7007,73 | 7059,30 | Iberian | 1596 |  |
| Natal | Brasil | 1490,30 | 9283,64 | Iberian | 1599 |  |
| Sao Luis | Brasil | 1421,74 | 7401,08 | European | 1612 |  |
| Belem | Brasil | 2553,99 | 9856,76 | Iberian | 1616 |  |
| Manaus | Brasil | 11477,50 | 8983,80 | Iberian | 1669 |  |
| Florianopolis | Brasil | 2273,92 | 6296,71 | Iberian | 1673 |  |
| Quillota | Chile | 440,10 | 6941,35 | Iberian | 1717 |  |
| Cuiaba | Brasil | 4569,88 | 5220,63 | Iberian | 1719 |  |
| Fortaleza | Brasil | 4022,93 | 8531,57 | Iberian | 1726 |  |
| San Nicolas de los Arroyos | Argentina | 677,40 | 5307,13 | Iberian | 1748 |  |
| Macapa | Brasil | 8041,99 | 9453,05 | Iberian | 1758 |  |
| Sincelejo | Colombia | 282,28 | 15750,26 | Iberian | 1776 |  |
| Rio Cuarto | Argentina | 18629,89 | 3471,73 | Iberian | 1786 |  |
| San Rafael | Argentina | 32150,71 | 4735,16 | Iberian | 1805 |  |
| Tandil | Argentina | 4864,86 | 4968,24 | Local | 1823 |  |
| Juiz de Fora | Brasil | 1442,68 | 9283,19 | Local | 1850 |  |
| Alagoinhas | Brasil | 757,00 | 8025,42 | Iberian | 1852 |  |
| Rosario | Argentina | 3885,80 | 5839,55 | Local | 1852 |  |
| Puerto Montt | Chile | 1675,33 | 6646,66 | European | 1853 |  |
| Montes Claros | Brasil | 3589,06 | 7341,54 | Local | 1857 |  |
| Sete Lagoas | Brasil | 540,46 | 4950,82 | Iberian | 1867 |  |
| Pocos de Caldas | Brasil | 549,93 | 5726,89 | Local | 1872 |  |
| Campo Grande | Brasil | 8133,59 | 4676,84 | Local | 1872 |  |
| Mar del Plata | Argentina | 1442,36 | 6251,53 | Local | 1874 |  |
| Resistencia | Argentina | 4657,96 | 7153,80 | Local | 1878 |  |
| Formosa | Argentina | 6421,66 | 8392,63 | Local | 1879 |  |
| Rio Branco | Brasil | 8891,39 | 7010,23 | Local | 1882 |  |
| Rio Gallegos | Argentina | 33534,01 | 5731,89 | Local | 1885 |  |
| Rawson-Trelew | Argentina | 4093,98 | 5073,44 | European | 1886 |  |
| Barreiras | Brasil | 7907,52 | 6833,68 | Local | 1891 |  |
| Santa Rosa | Argentina | 7640,95 | 3968,99 | Local | 1892 |  |
| Jequie | Brasil | 3246,59 | 7432,17 | Local | 1897 |  |
| Belo Horizonte | Brasil | 4615,90 | 8057,98 | Local | 1897 |  |
| Florencia | Colombia | 2390,68 | 16480,03 | Local | 1902 |  |
| San Carlos de Bariloche | Argentina | 5540,17 | 5017,91 | Local | 1902 |  |
| Mexicali | Mexico | 15701,16 | 4426,60 | Local | 1903 |  |
| Neuquen-Plottier-Cipolletti | Argentina | 21939,44 | 4680,38 | Local | 1904 |  |
| Anapolis | Brasil | 1533,80 | 5304,26 | Local | 1907 |  |
| Itabuna | Brasil | 434,78 | 11802,09 | Local | 1910 |  |
| Juazeiro do Norte | Brasil | 2007,81 | 8655,21 | Local | 1911 |  |
| Maraba | Brasil | 15228,40 | 7352,30 | Local | 1913 |  |
| Arapiraca | Brasil | 358,44 | 8315,38 | Local | 1924 |  |
| Goiania | Brasil | 3516,04 | 5829,99 | Local | 1933 |  |
| Dourados | Brasil | 4105,96 | 4604,92 | Local | 1935 |  |
| Governador Valadares | Brasil | 2354,84 | 7447,19 | Local | 1938 |  |
| Brasilia | Brasil | 10636,27 | 6991,63 | Local | 1960 |  |
| Ipatinga | Brasil | 2076,15 | 7873,15 | Local | 1964 |  |
| European corresponds to non-Iberic countries, Iberian corresponds to Spain and Portugal, Pre-hispanic corresponds to indigenous populations, and Local corresponds to native people after independency | | | | | |  |
|  |  |  |  |  |  |  |
|  |  |  |  |  |  |  |
|  |  |  |  |  |  |  |
