# Supplementary material for: Built environment profiles for Latin American urban settings: The SALURBAL study
Source: PLoS One. 2021 Oct 26;16(10):e0257528. doi: 10.1371/journal.pone.0257528 (PMC8547632; doi:10.1371/journal.pone.0257528)
Supplement: Attachment — Submitted filename: PONE-D-20-18493__review.doc [file pone.0257528.s011.doc]

The authors identify city profiles based on the built landscape and street design characteristics of cities in Latin America and evaluate the associations of city profiles with social determinants of health and air pollution. The manuscript is well-structured and contributes to studies related to morphological classifications of cities/urban areas and their associations with social aspects of health determinants. In addition, the manuscript also contributes to the literature on morphological classification of cities in Latin American context.

Some specific comments and suggestions are listed below:

1. A literature review of related past studies is missing and it is important to justify the methods used, to identify the gaps and to highlight the originality and contribution of this study to literature. More particularly, past studies using different pattern analysis to identify various profiles of cities should be highlighted. One such study is: *Southworth, M., & Owens, P. M. (1993). The evolving metropolis: Studies of community, neighborhood, and street form at the urban edge. Journal of the American Planning Association, 59(3), 271-287*. A more recent study is Peponis, J., Allen, D., Haynie, D., Scoppa, M., & Zhang, Z. (2007). Measuring the configuration of street networks: the spatial profiles of 118 urban areas in the 12 most populated metropolitan regions in the US. These are based on the classification of street connectivity of urban areas. What are the key metrics used previously? More regarding different measures should be cited. In addition, studies investigating the relationship between these patterns and health outcomes need to be cited too. Many previous studies are cited in the Discussion, but these should have been introduced in the Lit Rev.
2. In Table 1, why are no definitions provided for certain metrics used: i.e. directness? These already have been defined in the literature and indeed the authors define them later in the text.
3. Line 198: why is the threshold set to 25?
4. Table 2 shows the correlations of individual measures with social aspects of health determinants. I believe these need some discussion/explanation. For example, to me it is interesting and unexpected why intersection density is negatively correlated with air pollution whereas street density is positively correlated with the same measure.
5. Line 331: should be edited as “95% confidence interval” to be consistent with the note on Table 6.
6. The manuscript can be stronger if, in the Discussion, the authors discuss briefly the policy/planning implications of their findings, particularly the associations of urban landscape and street design with health aspects. Can any suggestions for urban planners be drawn based on the findings?
7. There are a couple sentences that need to be revised grammatically.

line 467: “of the fragmentation **an** density of…” *and?*

line 509: “Latin American…” should be *Latin America*
